# The impact of PTSD on risk of cardiometabolic diseases: a national patient cohort study in Norway

**DOI:** 10.1186/s12888-023-04866-x

**Published:** 2023-05-20

**Authors:** Grethe Emilie Roer, Lars Lien, Ingeborg Bolstad, Jan O. Aaseth, Dawit Shawel Abebe

**Affiliations:** 1 Norwegian National Advisory Unit on Concurrent Substance Abuse and Mental Health Disorders, Innlandet Hospital Trust, P.O. Box 104, NO-2381 Brumunddal, Norway; 2 Department of Nursing and Health Promotion, Oslo Metropolitan University, St. Olavs Plass, P.O. Box 4, NO-0130 Oslo, Norway; 3 Faculty of Social and Health Sciences, Inland Norway University of Applied Sciences, P.O. Box 400, NO-2418 Elverum, Norway; 4 Research Department, Innlandet Hospital Trust, P.O. Box 104, NO-2381 Brumunddal, Norway

**Keywords:** Posttraumatic stress disorder, Alcohol use disorder, Depression, Comorbidity, Cardiovascular diseases, Metabolic diseases, Diabetes mellitus, Epidemiology, Register data, Cohort study

## Abstract

**Background:**

Posttraumatic stress disorder (PTSD) is associated with cardiometabolic diseases, concurrent anxiety, alcohol use disorder and depression. The relationship between PTSD and cardiometabolic diseases are still unclear, and less is known about the effects of socioeconomic status, comorbid anxiety, comorbid alcohol use disorder and comorbid depression. The study, therefore, aims to examine the risk of developing cardiometabolic diseases including type 2 diabetes mellitus over time in PTSD patients, and to what extent socioeconomic status, comorbid anxiety, comorbid alcohol use disorder and comorbid depression attenuate associations between PTSD and risk of developing cardiometabolic diseases.

**Method:**

A retrospective, register-based cohort study with 6-years follow-up of adult (> 18 years) PTSD patients (*N* = 7 852) compared with the general population (*N* = 4 041 366), was performed. Data were acquired from the Norwegian Patient Registry and Statistic Norway. Cox proportional regression models were applied to estimate hazard ratios (HRs) (99% confidence intervals) of cardiometabolic diseases among PTSD patients.

**Results:**

Significantly (*p* < 0.001) higher age and gender adjusted HRs were disclosed for all cardiometabolic diseases among PTSD patients compared to the population without PTSD, with a variation in HR from 3.5 (99% CI 3.1–3.9) for hypertensive diseases to HR = 6.5 (5.7–7.5) for obesity. When adjusted for socioeconomic status and comorbid mental disorders, reductions were observed, especially for comorbid depression, for which the adjustment resulted in HR reduction of about 48.6% for hypertensive diseases and 67.7% for obesity.

**Conclusions:**

PTSD was associated with increased risk of developing cardiometabolic diseases, though attenuated by socioeconomic status and comorbid mental disorders. Health care professionals should be attentive towards the burden and increased risk that low socioeconomic status and comorbid mental disorders may represent for PTSD patients’ cardiometabolic health.

**Supplementary Information:**

The online version contains supplementary material available at 10.1186/s12888-023-04866-x.

## Introduction

Posttraumatic stress disorder (PTSD) [1], a severe sequela of traumatic experiences, characterized by symptoms such as flashbacks; nightmares; avoidance; a sense of “numbness” and emotional blunting; anhedonia and acute burst of fear or panic, frames a significant part of the population. Lifetime prevalence of PTSD is estimated to be 3.9%, with a variation of 2.1% in lower to low middle income countries and 5.0% in high-income countries [2]. PTSD, in turn, is associated with poorer physical health and health related quality of life [3] and lower life expectancy [4].

Although several systematic reviews and meta-analyses during the past years indicate increased risk for developing cardiometabolic diseases, e.g., cardiovascular diseases (CVD), cardiovascular risk factors such as type 2 diabetes mellitus (T2DM), and metabolic syndrome (MetS) in people with PTSD [5–7], a causal relationship has been difficult to establish [8]. The pathogenesis of cardiometabolic diseases in people with PTSD is complex, consisting of biological, psychosocial and behavioural factors (e.g., eating behavior, physical inactivity and smoking) [9]. For example, traumatic stress might impact eating behavior [10], that over time can cause adverse health consequences. Koenen, et al*.* [8] suggest at least five different causal structures explaining the statistical association between PTSD and cardiometabolic diseases; 1) a confounding bias explaining the relationship (e.g., trauma exposure and socioeconomic status (SES)); 2) a reverse causal association, i.e., myocardial infarction can lead to PTSD; 3) a one-way link between PTSD and cardiometabolic disease, i.e., PTSD causes cardiometabolic diseases; 4) a mediating factor explaining the association, e.g. smoking; or 5) a two-way association, i.e., scenario 2 and 3. However, although the majority of previous studies have found an association between PTSD and an increased risk of cardiometabolic diseases, Crum-Cianflone*, *et al*.* [11] found that PTSD symptoms in United States military personnel was not associated with coronary heart diseases after adjustment for depression and anxiety.

People with PTSD are often diagnosed with concurrent alcohol use disorder (AUD) [12], depressive disorders [13] and anxiety [14]. Estimated prevalence for co-occurring AUD range between 13% in Denmark to 55% in USA [14–16], while for co-occurring major depressive disorder and anxiety, the prevalence is estimated to be 52% and 49%, respectively [13, 14]. Both anxiety, AUD, and depressive disorders are in turn associated with increased risk of cardiometabolic diseases [17–19]. However, research on the effect of comorbid AUD on the risk of developing cardiometabolic diseases in people with PTSD is so far limited, and the majority of studied populations regarding impacts of comorbid depression on the association between PTSD and cardiometabolic diseases consist of war veterans and military service personnel. In addition, since the effect of depression and anxiety on the cardiovascular system seems comparable, Batelaan*, *et al*.* [20] suggests adjusting for anxiety when examining the effect of depression on the cardiovascular system.

As people with PTSD are a particularly vulnerable group for developing cardiometabolic diseases, which may negatively impact quality of life and life expectancy, knowledge about how health and disease develops over time, including possible risk factors for development of serious diseases, is of importance for developing measures to prevent diseases and early death. Confounding factors such as gender, increased age and SES appear to impact on health outcome [5, 21–23], and should thus be considered when studying health and diseases in a population. Moreover, mental disorders are associated with lower SES [24]. Large population-based retrospective cohort studies, investigating the occurrence of cardiometabolic diseases, including the effect of SES, comorbid anxiety, comorbid AUD and comorbid depression, in people with PTSD, are limited and there is a need for further exploration. The present study thus aims to answer the following research questions; 1) what is the risk of developing cardiometabolic diseases including T2DM over time in PTSD patients? and 2) to what extent do SES, comorbid anxiety, comorbid AUD and comorbid depression attenuate associations between PTSD and risk of developing cardiometabolic diseases?

## Methods

### Study design and population

**Fig. 1:**
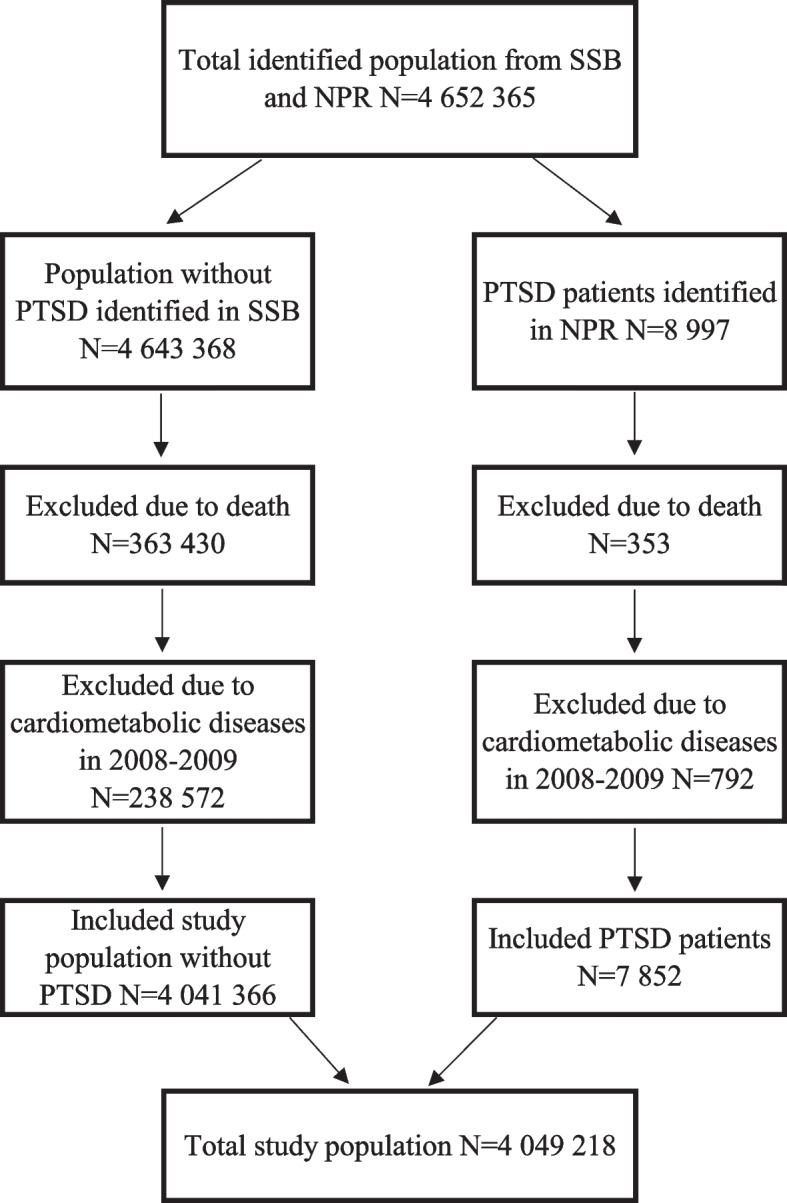
Study population flow chart

This is a register-based cohort study combining sociodemographic information from Statistics Norway and information on physical diseases and mental disorders obtained from the Norwegian Patient Registry (NPR). NPR holds data on all registered diagnoses obtained during contacts with specialist health care services. The unique number assigned to each person enables tracking the same patient over time from hospital to hospital, and analysing the data without concern of duplicates of cardiometabolic events. All diagnoses are based on the background of the International Classification of Diseases 10^th^ Revision (ICD-10) codes [1]. As illustrated in Fig. 1, the total identified study population consisted of all adults 18 years or older who were legal residents in Norway starting from January 1, 2008 to December 31, 2016 (*N* = 4 652 365). PTSD patients (*N* = 8 997) were identified during the preceding 2 years (2008–2009), and then followed for cardiometabolic comorbidity for 6 years, from January 1, 2010, through December 31, 2016. People who were registered as deceased (*N* = 363 783) during the study period (2008–2016) were excluded from analysis, and a two years washout period (2008–2009) was performed to exclude all individuals with cardiometabolic diseases (*N* = 792 with PTSD and *N* = 238 572 without PTSD).

### Exposure, explanatory and outcome variables

All diagnoses were received during outpatient and inpatient contacts with specialist healthcare between 2008 and 2016. We defined cardiovascular diseases, endocrine and metabolic diseases according to the ICD-10 codes [1]. The following dichotomous variables in Table 1 representing ICD-10 diagnostic categories were coded to specific disorders.

**Table 1:** ICD-10 codes and year of diagnosis for primary exposure variables, explanatory variables and outcome variables

	**ICD-10 Codes**	**Year of diagnosis**
**Primary exposure variable**		
PTSD	F43.1	2008–2009
**Explanatory variables**		
Anxiety	F40-F41	2008–2009
AUD	F10	2008–2009
Depression	F32-F33	2008–2009
**Outcome variables**		
Cardiovascular diseases		
Hypertensive diseases	I10-I15	2010–2016
Ischaemic heart diseases	I20-I25	2010–2016
Pulmonary heart diseases	I26-I28	2010–2016
Cerebrovascular diseases	I60-I69	2010–2016
Endocrine and metabolic diseases		
Diabetes mellitus	E10-E14	2010–2016
Obesity	E66	2010–2016
Metabolic disorders	E70-E90	2010–2016

### Covariates

Age and gender were used as covariate variables. The age variable (per 1. January 2008) was used as a continuous variable. Gender was coded 0 for males and 1 for females. We included being recipient of social benefits as an index for SES. According to the EU-scale, household poverty is defined as less than 60% of the annual median disposable equivalised household income after adjusted for the size of household members. A dummy variable (0 and 1) was constructed to indicate whether participants were defined as living in a household with poverty in each year. A sum score for all years was calculated with 0 being the lowest score (no years in poverty) and 9 being the highest sum score (in poverty during all years in the study). A higher sum score indicates more years with household poverty between 2008 and 2016.

### Statistical analysis

Before the analysis, a directed acyclic graph (DAG) [25] was created to identify and illustrate unobserved and adjusted variables, and causal pathways (Online Supplementary Fig. 1). The Cox proportional regression models were applied to estimate the risks of cardiometabolic diseases (event outcomes) among PTSD patients (an independent risk factor). Hazard ratios (HRs) with 99% confidence intervals (99% CIs) were reported, with calendar year as the underlying time axis. A stepwise regression was applied: model 1 presents age and gender adjusted HR estimates; HR estimates in model 2 adjusted for age, gender and recipient of social welfare (SES indicator); HR estimates in model 3 adjusted for age, gender, SES indicator and comorbid anxiety; HR estimates in model 4 adjusted for age, gender, SES indicator, comorbid anxiety and comorbid AUD; HR estimates in model 5 adjusted for age, gender, SES indicator, comorbid anxiety and comorbid depression; and HR estimates in model 6 adjusted for age, gender, SES indicator, comorbid anxiety, comorbid depression and comorbid AUD. Estimates were judged as statistically significant when *p*-values ≤ 0.01. The analyses were performed using Stata SE/16.

The proportional hazard assumptions were tested for each model on the basis of Schoenfeld residuals. Since some of the covariates did not often meet the proportional hazards assumption after fitting each model (“*estat phtest tests*”), interaction terms between time and time-variant covariates (age and SES), including specified stratification for gender in all models, were added.

To evaluate our findings against potential confounding factors, a sensitivity analysis was performed. The E-values for the association in the model adjusted for comorbid mental disorders found in our Cox regression analysis were calculated. The E-value represent the minimum strength of association between outcome and unmeasured confounding factors that would be required to explain away the association between the outcome variable and explanatory variables presented in the Cox regression. An E-value that is small relative to the HR would indicate that even weak confounding effects could explain the association, while a relatively large E-value suggests that the association is unlikely to be explained by confounding factors [26] (Online Supplement Table 1).

### Ethics

All study procedures were approved by the Norwegian Regional Committee for Medical and Health Research Ethics (ref: 17/26919–5).

## Results

### Description of the study population

A descriptive summary of the study population's characteristics is presented in Table 2. Among the total of 7 852 adults (> 18 years) registered with the diagnosis PTSD in the period 2008–2009, a higher number of women (*N* = 5 372, 68.4%) than men (*N* = 2 480, 31.6%). For the population with PTSD patients, the mean age was 37.8 years (SD = 11.5), and the mean for receiving social welfare (ranging from 0 to 9) 0.6 (SD = 1.2). For the population *without* PTSD, the mean age was 44.7 years (SD = 15.9), and the mean for receiving social welfare was 0.1 (SD = 0.4).

**Table 2:** Descriptive summary of the study population

	Population without PTSD (*N* = 4 041 366, 100%)	Population with PTSD (*N* = 7 852, 100%)
**Covariates and explanatory variables**		
Gender: N (%)		
Men	2 286 220 (56.6)	2 480 (31.6)
Women	1 755 121 (43.4)	5 372 (68.4)
Age (years): Mean (SD)	44.7 (15.9)	37.8 (11.5)
Anxiety: N (%)	33 181 (0.8)	1 411 (18.0)
AUD: N (%)	13 401 (0.3)	417 (5.3)
Depression: N (%)	48 586 (1.2)	2 477 (31.6)
Recipient of social welfare: Mean (SD)	0.1 (0.4)	0.6 (1.2)
**Event outcomes:** N (%)		
Hypertensive diseases	245 062 (6.1)	428 (5.5)
Ischaemic heart diseases	127 359 (3.2)	230 (2.9)
Pulmonary heart diseases	17 353 (0.4)	43 (0.6)
Cerebrovascular diseases	58 621 (1.5)	106 (1.4)
Diabetes mellitus	98 574 (2.4)	293 (3.7)
Obesity	45 949 (1.1)	344 (4.4)
Metabolic disorders	134 367 (3.3)	519 (6.6)

During 2008–2009, the proportion registered with comorbid mental disorders was higher among PTSD patients compared with the general population *without* PTSD. Compared with the population *without* PTSD, a smaller proportion of the population with PTSD patients was registered with hypertensive diseases, ischaemic heart diseases and cerebrovascular diseases during the period 2010–2016. A larger proportion of the population with PTSD patients was registered with pulmonary heart diseases, diabetes mellitus, obesity and metabolic disorders than the population *without* PTSD during the period 2010–2016.

### Risks of cardiometabolic diseases among PTSD patients and the effect of SES and comorbid mental disorders

Table 3 presents results (HR with 99% CI) from stepwise Cox regression models, showing HR for cardiometabolic diseases in PTSD patients adjusted for age and gender in model 1; age, gender and SES indicator in model 2; age, gender, SES indicator and comorbid anxiety in model 3; age, gender, SES indicator, comorbid anxiety and comorbid AUD in model 4; age, gender, SES indicator, comorbid anxiety and comorbid depression in model 5; and age, gender, SES indicator, comorbid anxiety, comorbid depression and comorbid AUD in model 6.

**Table 3:** Cox regression models showing adjusted HRs of cardiometabolic comorbidities among PTSD patients compared to the population without PTSD

Event outcomes	**Model 1** HR (99% CI)	**Model 2** HR (99% CI)	**Model 3** HR (99% CI)	**Model 4** HR (99% CI)	**Model 5** HR (99% CI)	**Model 6** HR (99% CI)
Hypertensive diseases	3.5 (3.1–3.9)	3.1 (2.7–3.5)	2.6 (2.3–2.9)	2.4 (2.1–2.7)	1.8 (1.6–2.1)	1.8 (1.6–2.1)
Ischaemic heart diseases	4.2 (3.6–5.0)	3.6 (3.1–4.3)	2.9 (2.5–3.5)	2.7 (2.3–3.2)	2.0 (1.7–2.4)	2.0 (1.7–2.4)
Pulmonary heart diseases	4.6 (3.1–6.8)	3.8 (2.6–5.7)	2.9 (1.9–4.3)	2.7 (1.8–4.0)	1.9 (1.3–2.9)	1.9 (1.2–2.8)
Cerebrovascular diseases	4.2 (3.3–5.4)	3.5 (2.7–4.5)	2.8 (2.1–3.6)	2.5 (1.9–3.2)	1.8 (1.4–2.4)	1.8 (1.4–2.3)
Diabetes mellitus	5.0 (4.3–5.8)	4.1 (3.5–4.7)	3.3 (2.8–3.8)	3.1 (2.6–3.6)	2.2 (1.9–2.6)	2.2 (1.9–2.6)
Obesity	6.5 (5.7–7.5)	5.0 (4.3–5.7)	3.6 (3.1–4.2)	3.4 (2.9–3.9)	2.1 (1.8–2.4)	2.0 (1.8–2.3)
Metabolic disorders	6.2 (5.5–6.9)	5.0 (4.5–5.6)	3.7 (3.3–4.1)	3.1 (2.8–3.5)	2.3 (2.1–2.6)	2.2 (2.0–2.5)

Model 1 = estimates adjusted for age and gender; model 2 = estimates adjusted for age, gender and recipient of social welfare (SES indicator); model 3 = estimates adjusted for age, gender, SES indicator and comorbid anxiety; model 4 = estimates adjusted for age, gender, SES indicator, comorbid anxiety and comorbid AUD; model 5 = estimates adjusted for age, gender, SES indicator, comorbid anxiety and comorbid depression; model 6 = estimates adjusted for age, gender, SES indicator, comorbid anxiety, comorbid depression and comorbid AUD. All HR estimates are statistically significant under *p*-value less than 0.001

Age and gender adjusted estimates in model 1 presents significantly higher HRs of all cardiometabolic diseases among PTSD patients compared to the population without PTSD, ranging from 3.5 (99% CI 3.1–3.9) for hypertensive diseases to 6.5 for obesity (99% CI 5.7–7.5). Although adjusting for SES and comorbid mental disorders in model 2–6 resulted in reductions of HR estimates of all cardiometabolic diseases, PTSD patients still had statistically significant higher HRs in all cardiometabolic diseases than the population without PTSD. E-values that were calculated for model 6 indicated relatively robust estimates of HRs (Online Supplement Table 1).

Compared to model 1, HR estimates in model 2 decreased for all cardiometabolic diseases; 11.4% for hypertensive diseases; 14.3% for ischaemic heart diseases, 17.4% for pulmonary heart diseases; 16.7% for cerebrovascular heart diseases; 18.0% for diabetes mellitus; 23.1% for obesity, and 19.4% for metabolic disorders.

Compared to model 2, HR estimates in model 3 decreased for all cardiometabolic diseases; 16.1% for hypertensive diseases; 19.4% for ischaemic heart diseases; 23.7% for pulmonary heart diseases; 20.0% for cerebrovascular diseases; 19.5% for diabetes mellitus; 28.0% for obesity, and 26.0% for metabolic disorders.

Compared to model 3, HR estimates in model 4 decreased for all cardiometabolic diseases; 7.7% for hypertensive diseases; 6.9% for ischaemic heart diseases; 6.9% for pulmonary heart diseases; 10.7% for cerebrovascular heart diseases; 6.1% for diabetes mellitus; 5.6% for obesity, and 16.2% for metabolic disorders.

The largest reduction in HR was observed in model 5 and 6. Compared to model 3, HR estimates in these models decreased for all cardiometabolic diseases; 30.8% for hypertensive diseases; 31.0% for ischaemic heart diseases; 34.5% for pulmonary heart diseases; 35.7% for cerebrovascular heart diseases; 33.3% for diabetes mellitus; respectively 41.7% and 44.4%, for obesity, and respectively 37.8% and 40.5% for metabolic disorders. Compared to model 5, HR estimates in model 6 only decreased for obesity and metabolic disorders, 4.8% and 4.3% respectively.

## Discussion

The main finding in this large retrospective registry-based population study is that PTSD patients had increased risk of developing cardiometabolic diseases, compared to the general population without PTSD. Adjusted for SES and comorbid mental disorders, the association was attenuated, strongest by comorbid depression.

Our results confirm previous findings of associations between PTSD and increased risk of developing cardiometabolic diseases [5–7]. The theory of *allostatic load* [27]*,* which refers to the cumulative burden of chronic stress and stressful life events, can contribute to the understanding of the association between PTSD and cardiometabolic diseases. Allostatic load is associated with increased risk of several cardiometabolic diseases, and early traumatic life experiences have been associated with high levels of allostatic load in adulthood [28]. If environmental challenges exceed the individual's ability to cope, there is an *allostatic overload*, which might involve consequences such as low SES, poor sleep, physical inactivity, smoking, alcohol consumption and unhealthy eating [28], which in turn can lead to poor health outcomes. For example, in a recent qualitative study [10], trauma exposed inpatients described how traumatic stress impacted their eating behaviors, and for some, consumption of food and snacks with high amounts of sugar, salt and saturated fat.

Furthermore, traumatic experiences can cause several physiological and somatic responses, which in turn can influence the developing of cardiometabolic diseases. Stress affects secretion of stress hormones and thereby peripheral organs, especially the cardiovascular and immune systems, i.e. via neurotransmitters and hormones in the hypothalamus–pituitary–adrenal axis (HPA axis) and the sympathetico-adrenal-medullary system (SAM system) [29]. Chronic stress appears to affect metabolic processes through oxidative stress in fat cells and the cells' mitochondria. This might lead to increased visceral fat deposits [30]. Increased inflammatory cytokine levels in PTSD patients has also been found [31].

The attenuating effect of SES on the association between PTSD and the risk of developing cardiometabolic diseases supports previous reports showing that the risk of developing cardiometabolic diseases such as CVD and T2DM increases with lower SES [21]. Long-term social assistance recipients can limit life opportunities and income trajectories [32], and lead to poor health outcomes and social health inequities [23], which should be taken into account by the healthcare providers and policy makers.

Furthermore, results from previous research suggests comparable effects of depression and anxiety on the risk of cardiovascular diseases [20]. Surprisingly, our results documented a larger attenuating effect of comorbid depression than comorbid anxiety on the risk of all cardiometabolic diseases in PTSD patients. However, according to the ICD-10, PTSD is categorized as an anxiety disorder. Since symptoms of anxiety disorders and PTSD can overlap, this may explain why depression showed a greater effect than anxiety in this current study.

The attenuating effect of comorbid AUD on the association between PTSD and the risk of developing cardiometabolic diseases has been less studied, although the consequences of long-term alcohol use on the cardiovascular system, e.g. hypertension, cardiac arrhythmia, cardiomyopathy and heart failure are well established [33]. However, PTSD patients with comorbid AUD have also been shown to have poor access and use of healthcare services [34, 35], which might explain the increased risk of cardiometabolic diseases.

Our findings of an attenuating effect of comorbid depression are in line with previous studies. Earlier research has documented attenuating effects of comorbid depression in several cardiometabolic diseases among people with PTSD [36–38]. Furthermore, biological mechanisms in depression, including dysregulation of the HPA-axis function [39], and increased levels of pro-inflammatory cytokines [40], might explain the increased risk of developing cardiometabolic diseases. Genetic mechanisms are possible underlying factors for the development of both depression and cardiovascular diseases [41].

Additionally, the attenuating effect of comorbid mental disorders might both be related to confounding behavioral factors such as physical inactivity, poor dietary habits and smoking [42–46], while in turn can increase the risk of cardiometabolic diseases including T2DM [47].

### Strengths and limitations

There are several strengths in this large population-based cohort-study. Firstly, the study includes data about all registered cases of cardiometabolic diseases across two large groups over six years, which thus brings adequate statistical power to detect differences. Secondly, the coverage of healthcare services and the quality of health records in Norway is considered to be high [48], and clinical set diagnosis in the NPR are found to be accurate and consistent compared to research-based diagnosis of severe mental disorders [49]. The NPR data used in this study are therefore considered to be highly reliable. Thirdly, clinically set diagnosis from specialist care may, or may not, be reliable, but systematic differences between the populations are nevertheless unlikely, making the relative estimates valid.

There are, however, some challenges in the present study that are important to note. The e-values calculated for model seven should be interpreted with caution because they do not necessarily reflect reality. Further, the registries do not have information about the type of traumatic exposure, psychotherapy, medication use, volume of health care utilization (i.e. number of clinic encounters per month or year), or health behavior (e.g., eating behavior, nicotine dependence and physical activity level) that may constitute confounding factors; information about severity of AUD (e.g., volume and frequency of consumption); information about the onset and duration of problematic alcohol consumption; or information about the time where diagnosis of PTSD and comorbid mental disorders were set. There is a possibility to adjust for all F-codes (00–99) in the ICD-10 as an indicator for volume of healthcare utilization, however due to concerns for overadjustment, we did not. Further, a worryingly large proportion of trauma-exposed people with PTSD symptoms do not seek help and are more likely to use alcohol as self-medication [50]. In addition, misdiagnosis and under diagnosis of PTSD is not uncommon [51]. Finally, use of registry-data from NPR might promote a risk of misclassification.

### Implications and further research

The results of this present study highlight some important implications that could be relevant for clinical practice and policy development practice. As this study documented higher risk of cardiometabolic diseases among PTSD patients compared to the population without PTSD, this suggests a need for somatic health in PTSD patients to be given high priority by healthcare professionals, and that screening for somatic health should be considered as a routine part of the follow-up of PTSD patients to expose cardiometabolic risk factors and diseases and be able to start early preventive interventions. Furthermore, healthcare professionals should be aware of the additional burden these comorbid mental disorders may represent for PTSD patients' cardiometabolic health, and consider offering concomitant treatment of the disorders. In addition, with a political goal of levelling out social inequalities in health, the authorities should be attentive to, and take seriously, the increased risk that low SES may entail for cardiometabolic health in vulnerable groups such as PTSD patients. The complexity of the association between PTSD and cardiometabolic risk factors and diseases warrants future research to explore the effects of possible confounding factors such as health behavior (e.g., diet, physical activity, alcohol use and smoking).

## Conclusion

The current study shows that PTSD was associated with increased risk of developing cardiometabolic diseases, and that SES and comorbid mental disorders attenuated the risk. In other words, low SES and comorbid mental disorders in PTSD patients were associated with increased risk of developing cardiometabolic diseases. This implies that social inequalities in health among vulnerable populations like PTSD patients should be targeted.

## Supplementary Information

**Additional file 1:** **Supplemental material Figure 1.** Directed acyclic graph for the association between PTSD and cardiometabolic diseases. **Supplemental material Table 1.** E-values for the HRs of cardiometabolic comorbidities among PTSD patients compared to the population without PTSD.

## Data Availability

The data that support the findings of this study are available from Statistics Norway and Norwegian Directorate of Health for the Norwegian Patient Register, but restrictions apply to the availability of these data, which were used under license for the current study, and so are not publicly available. Data are however available from the corresponding author upon reasonable request and with permission of Statistics Norway and Norwegian Directorate of Health for the Norwegian Patient Register.

## Abbreviations

AUD: Alcohol use disorder
CI: Confidence Interval
CVD: Cardiovascular diseases
DAG: Directed acyclic graph
HR: Hazard Ratio
ICD-10: International Classification of Diseases 10^th^ Revision
MetS: Metabolic syndrome
NPR: Norwegian Patient Registry
PTSD: Posttraumatic stress disorder
T2DM: Type 2 diabetes mellitus
SES: Socioeconomic status
