# Supplementary material for: The impact of PTSD on risk of cardiometabolic diseases: a national patient cohort study in Norway
Source: BMC Psychiatry. 2023 May 20;23:349. doi: 10.1186/s12888-023-04866-x (PMC10200052; doi:10.1186/s12888-023-04866-x)
Supplement: Supplementary file 1 — Additional file 1: Supplemental material Figure 1. Directed acyclic graph for the association between PTSD and cardiometabolic diseases. Supplemental material Table 1. E-values for the HRs of cardiometabolic comorbidities among PTSD patients compared to the population without PTSD. [file 12888_2023_4866_MOESM1_ESM.docx]

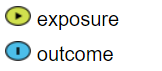
**Supplemental material figure 1.** Directed acyclic graph for the association between PTSD and cardiometabolic diseases.


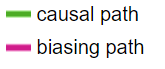
**
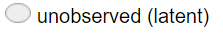
**
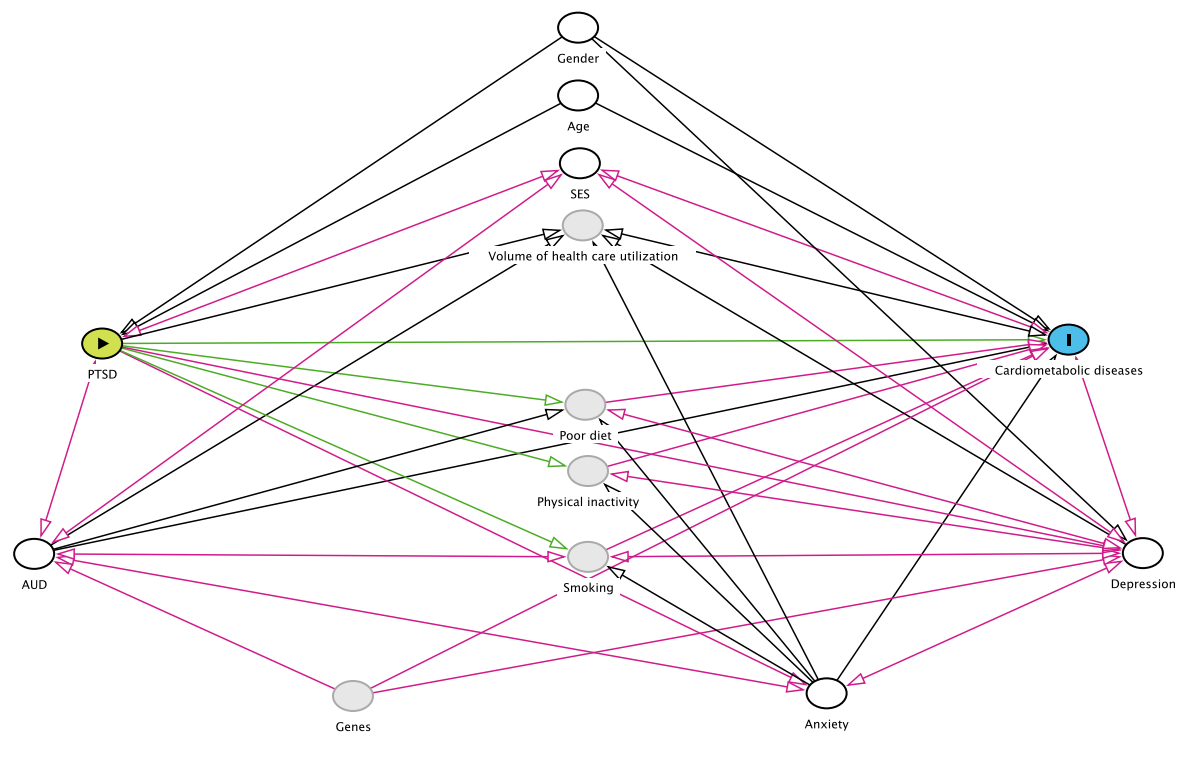


Further explanation about the directed acyclic graph could be found at [http://dagitty.net/].

**Supplemental material table 1.** E-values for the HRs of cardiometabolic comorbidities among PTSD patients compared to the population without PTSD

| Event outcomes |  | | **Model 6** | |
| --- | --- | --- | --- | --- |
|  |  |  | HR | CI* |
| Hypertensive diseases |  |  | 2.38 | 2.17 |
| Ischaemic heart diseases |  |  | 2.59 | 2.29 |
| Pulmonary heart diseases |  |  | 2.44 | 1.79 |
| Cerebrovascular diseases |  |  | 2.35 | 1.93 |
| Diabetes mellitus |  |  | 2.84 | 2.56 |
| Obesity |  |  | 2.64 | 2.39 |
| Metabolic disorders |  |  | 2.86 | 2.65 |

Model 6 = estimates adjusted for age, gender, SES indicator, comorbid anxiety, comorbid depression and comorbid AUD. *Lower bound of the 95% CI.
